# Fabry–Pérot modes associated with hyperbolic-like dispersion in dielectric photonic crystals and demonstration of a bending angle sensor at microwave frequencies

**DOI:** 10.1038/s41598-020-67965-9

**Published:** 2020-07-06

**Authors:** R. Rachel Darthy, C. Venkateswaran, V. Subramanian, Zhengbiao Ouyang, N. Yogesh

**Affiliations:** 1 Department of Nuclear Physics, School of Physical Sciences, University of Madras, Chennai, 600025 India; 2 Microwave Laboratory, Department of Physics, Indian Institute of Technology Madras, Chennai, 600036 India; 3 Terahertz Technical Research Center, College of Physics and Optoelectronic Engineering, Shenzhen University, Shenzhen, 518060 China

**Keywords:** Photonic crystals, Photonic devices

## Abstract

The dispersion properties of metamaterials and photonic crystals (PhCs) lead to an intensive research in the development of cavity resonators for the confinement of electromagnetic (e-m) radiation. In this work, we investigate the formation of Fabry–Pérot (FP) modes associated with hyperbolic-like dispersion (HLD) regimes in two-dimensional dielectric PhCs. Conventionally, FP modes are formed using an optical etalon, in which electromagnetic (e-m) waves reflecting from a partially reflecting mirror separated by a distance can interfere constructively and form a resonating mode. The FP mode observed in dielectric PhCs is formed due to the interference of cylindrical wavefronts inside the PhC interface at HLD frequencies. The FP modes in PhCs are surface localized, in which maxima/minima of the electric field lies along the air–PhC interface as a standing wave pattern and decays in air medium. Projected bandstructure, Eigen Frequency Contours (EFC), phase and group index calculations are carried out to explain the formation of FP modes in PhCs under different coupling cases. By varying the PhC dimension, FP modes with different spatial profiles are witnessed and the role of source position in exciting specific mode is demonstrated. The observed FP modes in PhCs are compared with the FP mode in an ideal indefinite slab. Based on the FP resonance in PhCs, a sensing device capable of detecting a bending angle less than $0.05^\circ$ is demonstrated numerically. The FP modes in PhCs are scalable to other parts of e-m spectra so that the bending angle sensing can be extendable to terahertz and optical domains.

## Introduction

Confining electromagnetic (e-m) radiation is one of the primary research objectives of photonics and a cavity resonator is a simple device to accomplish it, with features such as high quality factor, small mode-volumes, switching, filtering and so on. For example, a pair of mirrors separated by a distance can support selective standing wave resonance also known as Fabry–Perot (FP) resonance playing an inevitable part in spectroscopic techniques and resonant light-matter interactions. Instead of mirrors as a separate component, interface of dielectric/magnetic materials can act as a reflecting surface depending on the strength of dielectric permittivity (ε_r_) and magnetic permeability (μ_r_), and it can support FP modes in varieties of dielectric resonators. A rectangular bar of silicon nanowire and dielectric nano ribbons are a few examples for supporting FP modes^1,2^.

The arrival of artificial e-m structures such as photonic crystals (PhCs)^3^ and metamaterials (MTMs)^4^ revolutionizes the FP resonator’s development and overcomes many limitations of conventional FP resonators. Photonic crystals are the wavelength comparable periodic dielectric/magnetic constituents arranged in one-, two-, and three-dimensions offer bandgap for e-m radiation, and exhibit anomalous dispersion such as negative refraction, self-collimation and ultra-high divergence^3,5^. On the other hand, MTMs are sub-wavelength periodic structures whose constitutive parameters such as ε_r_ and μ_r_ can have negative values, and they exhibit novel e-m phenomena such as negative refraction, double-focusing, sub-wavelength imaging, cloaking and reversal of Doppler shit^4,6^. The PhC multilayer can act as a mirror and it can inhibit spontaneous emission at bandgap frequencies^7,8^. Hence the FP microcavity made of a PhC can enhance laser efficiency^7^. Similarly the role of MTMS in miniaturization of FP resonators is crucial, as miniaturized MTMs provide desired reflectance characteristics from microwave to visible frequencies^9,10^.

Recently much attention has been paid on the exploration of dispersion characteristics for the design of resonators supporting FP modes, especially the hyperbolic dispersion^11–13^. When the principal components of ε_r_ and μ_r_ tensors of an anisotropic medium have negative signs, the dispersion relation will trace out a hyperbolic eigenfrequency contour (EFC) in a wavevector plane, and such a medium is known as hyperbolic metamaterial (HMM) or an indefinite medium^14–18^. There are many artificial e-m structures are available for the realization of indefinite medium such as layered metal-dielectric systems^19^, multilayered fishnet structures^20^, nanorod arrays^21^, graphene MTM^22^, metasurfaces^23^, liquid crystals with silver nanoparticles^24^, transmission lines^25^ and photonic hypercrystals^26^.

Since hyperbolic EFC is opened, an indefinite medium can support large wavevectors so that FP resonator made of an indefinite medium can confine e-m modes with ultra-small mode volumes. Moreover, an indefinite medium can provide total-internal reflection condition for small critical angle due to large dielectric constant values^11,13^. It is also reported that FP resonator made of an indefinite medium follows anomalous scaling law, in which different resonator sizes support same e-m frequency. Apart from 3-D FP resonator^11^, planar type magnetic hyperbolic cavity exhibiting FP resonance is also reported recently^13^.

In this work, we explore the anomalous dispersion characteristics of two-dimensional dielectric PhCs^27–32^, especially the hyperbolic-like dispersion (HLD) regimes for the investigation of e-m mode confinement. A PhC made of all-dielectric constituents can support various dispersion regimes ranging from isotropic positive medium response (circular EFC) to left-handed behaviour (circular EFC but with negative effective index) ^27,29^ including an indefinite medium response (HLD-like EFCs)^30^. All-angle negative refraction without negative index was a typical example of an indefinite medium response of a dielectric PhC at first band frequencies^30^. Similarly, the HLD regime at higher-photonic bands can show anomalous refractive behaviour, and is capable of providing internal reflection condition suitable for FP mode formation as that of an ideal indefinite medium. However, it should be stressed that unlike the HMM or the examples of indefinite media discussed above, dielectric PhC does not have negative permittivity plasmonic material in it. The HLD regimes in dielectric PhCs are manifested from strong anisotropy and periodic modulation of dielectric elements^29^. Moreover, the assignment of constitutive parameter such as effective refractive index for a dielectric PhC is limited to stringent effective medium conditions^31,32^.

In the present work, we investigate the FP mode associated with HLD regimes of two-dimensional dielectric PhC at microwave frequencies through photonic bandstructure calculations, ray tracing, and refractive index calculations. Full-wave e-m computations are carried out to reveal the refraction and interference pictures on the formation of FP mode. By varying the PhC slab’s dimensions, we have observed the interesting spatial profiles of FP modes and we have also found that the role of e-m source position is important in exciting these modes. The FP mode in PhCs at HLD regimes is compared with the FP mode in an ideal indefinite medium. Finally, we demonstrate the application aspect of FP modes in sensing the bending angle and curvature of PhCs at microwave frequencies.

## Results

### Observation of FP Modes and HLD regimes in dielectric PhCs

**Figure 1:**
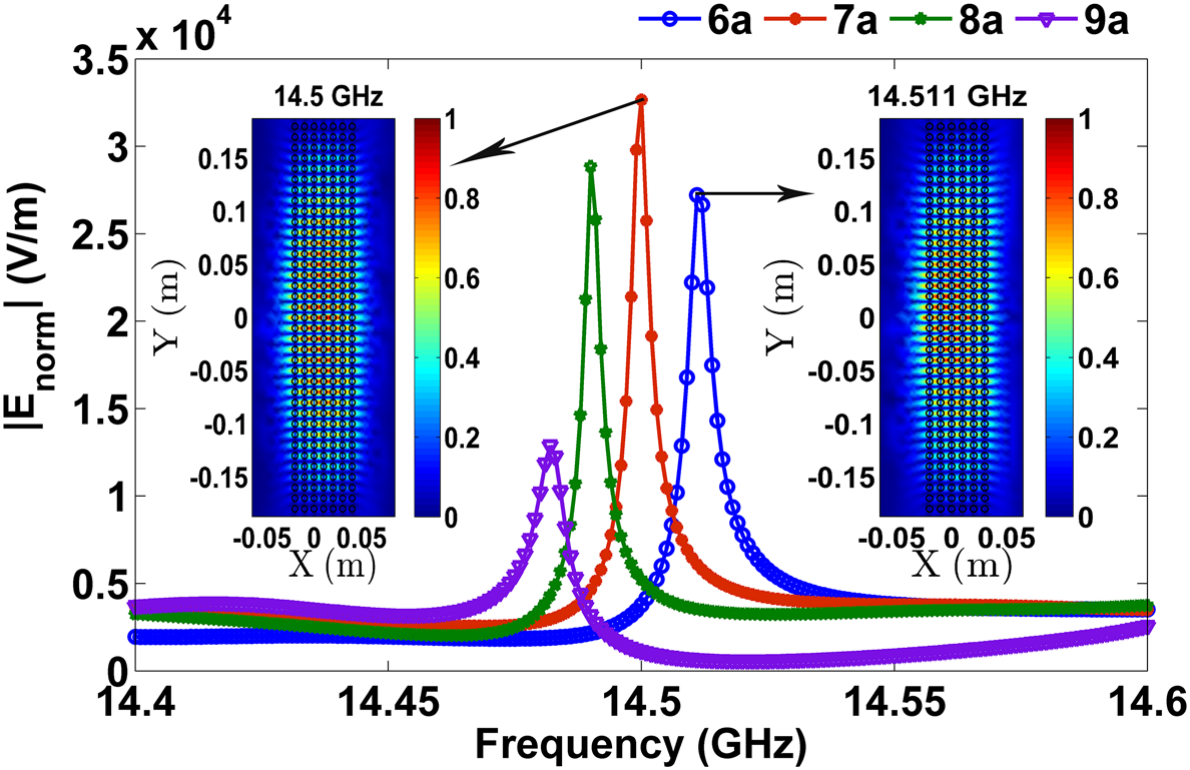
FP resonance spectra for various thicknesses of a glass PhC. Inset shows E_norm_ pattern at 14.511 GHz and 14.5 GHz for PhC thicknesses of 6*a* and 7*a*, respectively.

**Figure 2:**
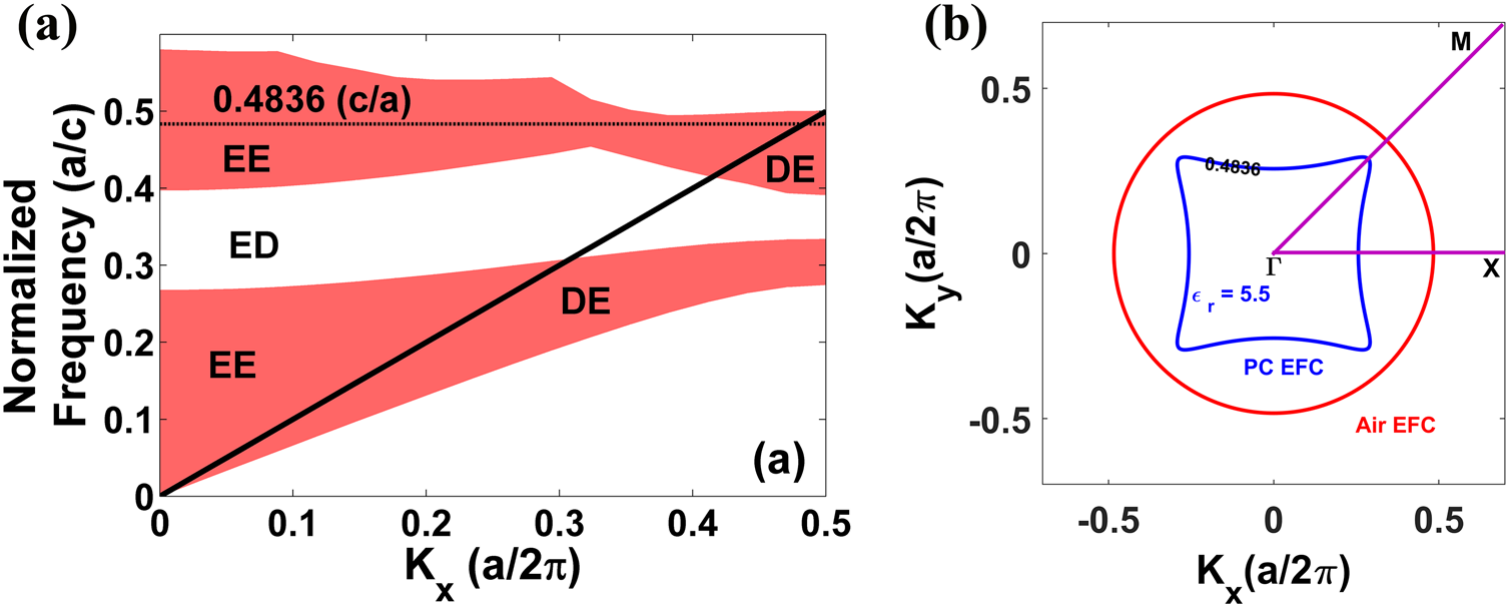
(**a**) Projected bandstructure of glass PhC for TE polarization mode. The dashed line corresponds to the normalized frequency of 0.4836(c/*a*). Solid line shows the light line. The expansion for ED, DE and EE modes are given in the main text. (**b**) EFC contour plot for air and glass PhC at 0.4836(c/*a*). Here $\Gamma ,X,M$ are the highest symmetry points of irreducible Brillouine zone of a square lattice PhC.

The square lattice PhC formed by the periodic arrangement of circular glass rods of radius $r = 0.3a$ in an air background is considered. Here ‘*a*’ is the lattice constant taken to be 1 cm. Glass has the relative dielectric permittivity of 5.5. When the transverse electric (TE) point source is excited near a glass PhC slab of various thicknesses, FP modes are formed as shown in Fig. 1. When the thickness of the PhC slab is increased from 6*a* to 9*a*, FP resonance is red-shifted linearly from 14.511 to 14.482 GHz as shown in Fig. 1. Similar to the conventional FP resonator, thickness decides the standing wave resonance in dielectric PhCs. It is also noticed that the mode pattern is localized on the surface, where the maxima/minima of electric field is localized along *y*-direction at the PhC-air interface, and the outside PhC, it decays in the air along the *x*-direction. The localization of mode on surface is well-studied phenomenon in PhCs^33,34^ and by harnessing the surface of PhCs, one can engineer characteristic e-m modes suitable for various applications^35–38^. In the present work, we investigate how the anomalous dispersion of PhCs plays major role in the formation of FP modes. Especially the observed mode suggests that the refracted wavefronts are interfering constructively inside the PhC depending on the thickness of the PhC. To verify the formation of FP modes, the projected band structure and EFCs are plotted in Fig. 2.

Figure 2a shows the TE mode projected bandstructure of a glass PhC. In the projected band structure, various modes are designated as follows; (1) modes which are extended (**E**) in air and extended (**E**) in PhC, are called as **EE** modes. These modes are propagating modes. (2) Modes which are decayed (**D**) in the air but extended (**E**) in PhC are called as **DE** modes. From Fig. 2a, it is observed that DE modes fall below the light line. (3) Modes which are extended (**E**) in air but decayed (**D**) in PhCs are called as **ED** modes and these modes are forbidden as they correspond to the bandgap regime of the PhC^33,34^. It is observed from Fig. 2a that the FP mode for a glass PhC at 0.4836(c/*a*) shares the regimes both above (EE regime) and below the light line (DE regime). This aspect indicates that when a point source is excited from air medium, e-m wave is coupled strongly for almost all range of *k*_*x*_ values because mode above the light line will be coupled effectively to the PhC. On the other hand, near band-edge, i.e., at higher *k*_*x*_ values, this mode [0.4836(c/*a*)] falls below the light line. Hence the refracted cylindrical wavefront at a higher incident angle is expected to be reflected inside the PhC.

In Fig. 2b, EFCs are plotted for air and glass PhC at 0.4836(c/*a*). Firstly, it is found that the shape of the EFC at 0.4836(c/*a*) is hyperbolic-like. It should be noted that unlike a trivial hyperbola, hyperbolic regimes in PhCs are closed due to the periodic modulation of dielectrics in both *x* and *y* directions. Secondly, it is observed that the size of the air EFC is larger than the size of the PhC EFC at 0.4836(c/*a*), i.e., air EFC includes PhC EFC. Hence an e-m wave incident from the air for a broad range of incident angles is effectively coupled to the PhC EFC. From the projected mode and EFC plot, we come to know that FP mode observed in glass PhC corresponds to the HLD EFC, and in case of a glass PhC, most of the HLD EFC is available for coupling.

### Observation of FP modes in PhCs with strong anisotropy

**Figure 3:**
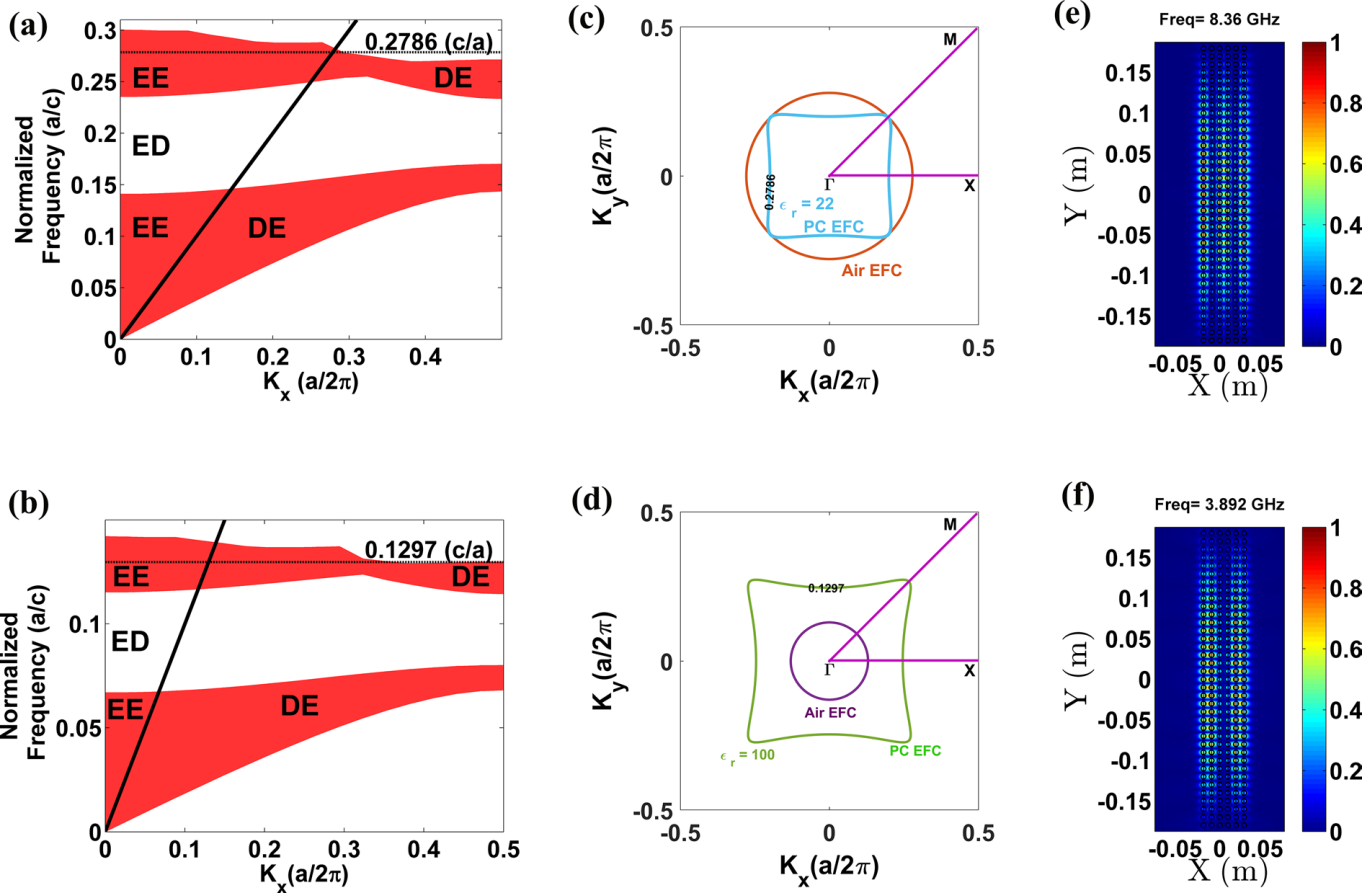
(**a**,**b**) TE mode projected bandstructures of PhC2 and PhC3, respectively; (**c**,**d**) EFC contours plot of PhC2 and PhC3 and (**e**,**f**) FP mode profiles of PhC2 and PhC3 at 0.2786(c*/a*) and 0.1297(c/*a*), respectively.

Apart from glass PhC, FP modes associated with HLD regimes in dielectric PhCs are also verified in the case of a PhC with strong anisotropy. To show this, two different PhCs with dielectric contrasts 22:1 (PhC2) and 100:1 (PhC3) are considered. Except dielectric constant values (PhC2 rods $\varepsilon_{r} = 22$ and PhC3 rods $\varepsilon_{r} = 100$), all other parameters such as lattice type, lattice constant, radius of atom are same as that of the glass PhC. The projected bandstructure, EFC plot and FP mode profiles are shown in Fig. 3. In the case of strong anisotropy also, FP modes are associated with HLD regimes but with different coupling behavior. In the case of PhC2, the HLD EFC is intersecting with air EFC (Fig. 3c) whereas, in the case of PhC3, the HLD EFC is large than air EFC (Fig. 3d). However, both the cases show FP resonance at 0.2786 (c/*a*) and 0.1297 (c*/a*), as shown in Fig. 3e,f, respectively. Hence under three different coupling cases, i.e. (1) air EFC is larger than PhC EFC, (2) air EFC and PhC EFCs are intersecting and (3) PhC EFC is larger than air EFC, the formation of FP mode at HLD regime is witnessed. From this observation, it is verified that the observed FP mode is the generalized characteristic of a dielectric PhC associated with the HLD regime and therefore analyzing their refractive index profiles are further essential.

### Role of refractive index in the formation of FP mode

To gain insight on the refraction picture at FP resonance, phase index (*n*_*p*_), group index (*n*_*g*_) and the angle between phase and group velocity vectors ($\phi$) are computed for three different PhCs with respect to incident angle variation. The phase index (*n*_*p*_) is defined as $n_{p} = {\text{sgn}} \, (\vec{k} \cdot {\vec{v}_{g}} ) \, \frac{{\left| {\vec{k}_{phc} } \right|}}{{\left| {\vec{k}_{air} } \right|}}$^39^, where $\vec{k}_{phc}$ is the wavevector of the PhC, $\vec{k}_{air}$ is a wavevector of the air medium and ${\text{sgn}} \, (\vec{k} \cdot {\vec{v}_{g}} )$ is the sign function between the scalar product of wave and group velocity ($\vec{v}_{g}$) vectors. The group velocity vector can be defined as $\vec{v}_{g} = \nabla \omega_{k}$ , where ω_k_ are the angular dispersion frequencies. The group index (*n*_*g*_) is calculated as $n_{g} = \frac{1}{{\nabla \omega_{k} }} = \frac{1}{{\left| {\vec{v}_{g} } \right|}}$.

**Figure 4:**
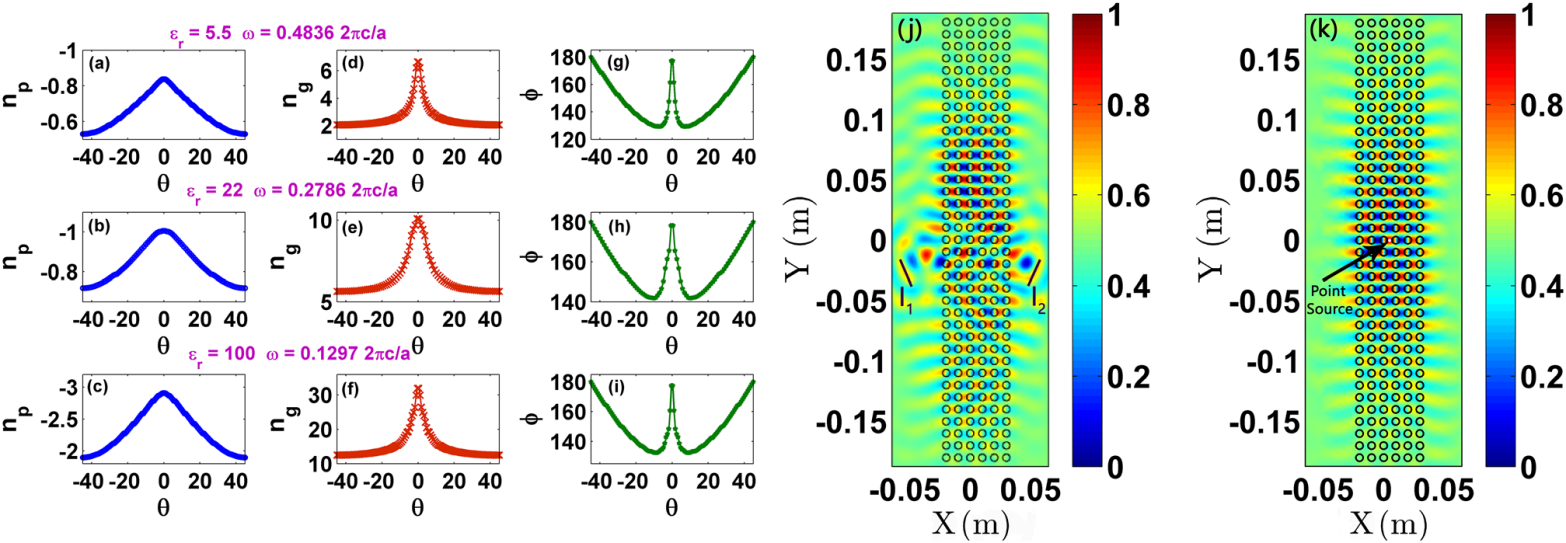
(**a**–**c**) Phase index (*n*_*p*_), (**d**–**f**) group index (*n*_*g*_) and (**g**–**i**) angle plot ($\phi$) between phase and group velocity vectors with respect to incident angle variation at FP resonance frequencies of glass PhC, PhC2 and PhC3 respectively. (**j**) E_z_ field map for a two line sources (marked as l_1_ and l_2_) incident on a glass PhC with an incident angle of 26° and − 26° at 14.511 GHz. (**k**) E_z_ field map at 14.511 GHz for TE point source placed inside the glass PhC.

Figure 4 shows *n*_*p*_, *n*_*g*_ and $\phi$ plots for glass PhC, PhC2 and PhC3 at their respective FP mode resonances. The $\phi$ plot (Fig. 4g–i) reveals that at HLD regime, the phase and group velocity vectors follow obtuse angle behaviour, in which $\vec{k} \cdot {\vec{v}_{g}} < 0$. Therefore, HLD regime exhibits mixed refractive behaviour, in which the phase index is negative (Fig. 4a–c) but the group index is positive (Fig. 4d–f). Mixed refraction is one of the salient characteristics of an indefinite medium. From Fig. 4, it is noted that the *n*_*p*_ and *n*_*g*_ at HLD frequencies have strong functional dependence on the incident angle, and PhC is strongly dispersive. Suppose one considers isotropic, homogeneous and non-magnetic dielectric medium with refractive index *n* in air background, the critical angle (*θ*_*c*_) condition for total internal reflection from denser to rarer medium is read as $\theta_{c} = \sin^{ - 1} \left( {{1 \mathord{\left/ {\vphantom {1 n}} \right. \kern-\nulldelimiterspace} n}} \right)$. For example, isotropic media with $n_{1} = 1.897$ and $n_{2} = 2.345$, correspond to $\theta_{c1} = 31.1^{ \circ }$ and $\theta_{c2} = 25.24^{ \circ }$, respectively. This implies that a conventional medium with high refractive index shows total internal reflection at lower critical angles. Similar to an isotropic medium, *θ*_*c*_ cannot be directly evaluated for PhCs owing to its strong spatial dispersion behaviour. However, PhC can provide rich spatial distribution of *n*_*g*_ with sufficiently high values for various incident angles at HLD EFCs. This kind of distribution cannot be expected for circular EFC regimes, as they indicate isotropic nature. Therefore, one can anticipate that the internal reflection of refracted rays inside the PhCs could be observable for all three HLD EFC cases (Figs. 2b, 3c,d), and if these internally reflected rays interfere with each other which depends on the thickness of PhC, one can expect the formation of FP modes. To verify this notion, numerical demonstration of interference of e-m wave inside the PhC is carried out in Fig. 4j,k with the following idea.

A point source emits an e-m wave with all possible angles of incidence. If one takes two beams with opposite incident angles and excite a glass PhC, then it is possible to verify whether they are interfering to form an FP mode or not as proposed. In Fig. 4j, E_z_ field map at 14.511 GHz is shown for a two line sources (marked as l_1_ and l_2_ in Fig. 4j) incident on a glass PhC with incident angles of 26° and − 26°. To create a phase difference between two beams, the distance between the line source and PhC is kept differently for two sources. It is evident that the interference of wavefronts from two-beams, forms FP mode in glass PhC at HLD frequency. To complete the demonstration, in Fig. 4k E_z_ field map at 14.511 GHz is shown for a point source placed inside the PhC. As expected, FP mode is formed at HLD frequency.

### Resonator’s size variation: observation of higher order FP modes in PhCs

**Figure 5:**
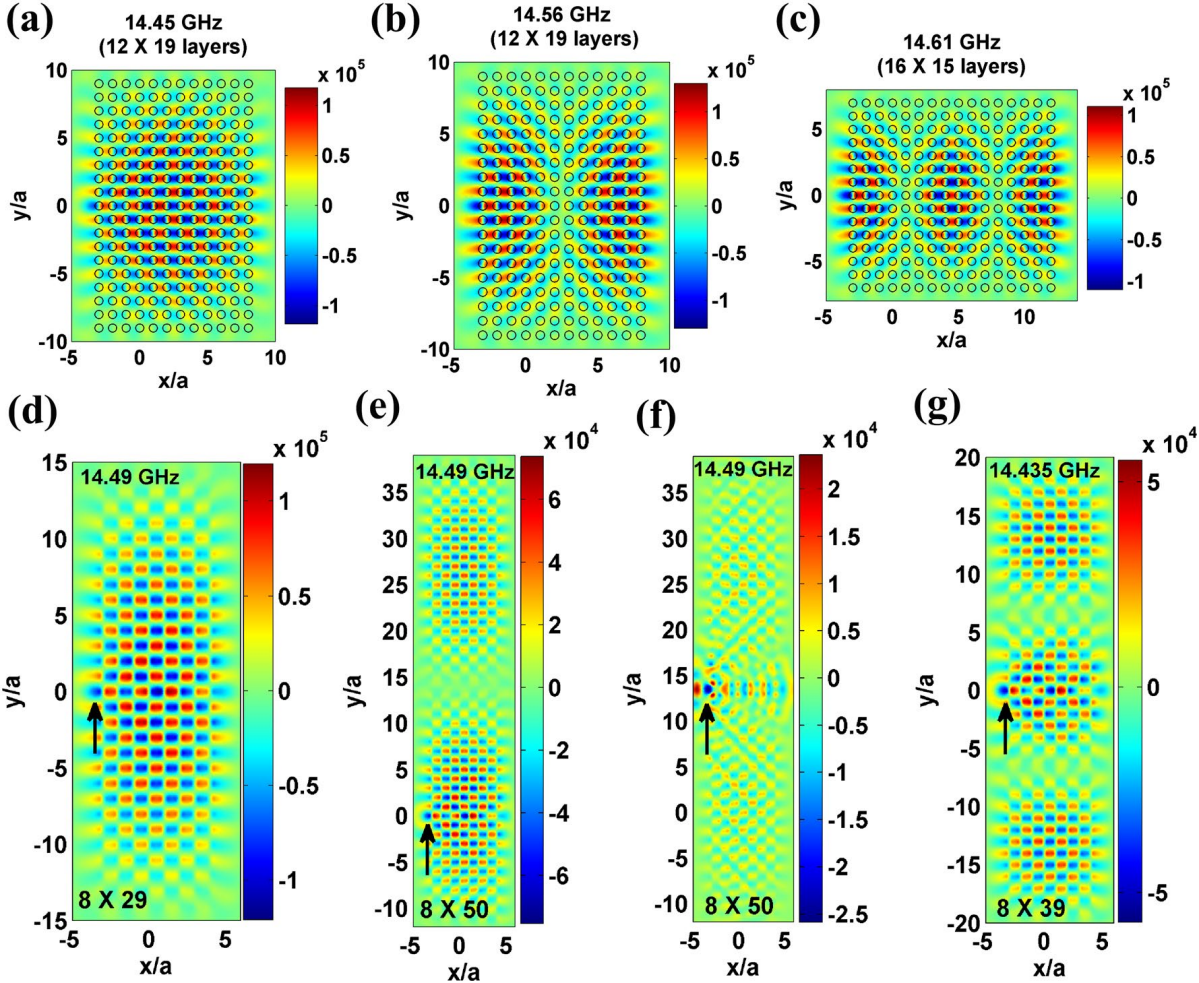
(**a**–**c**) FP mode profiles at 14.45 GHz, 14.56 GHz and 14.61 GHz in a PhC slab of dimensions $12 \times 19$, $12 \times 19$, and $16 \times 15$ layers, respectively. (**d**–**g**) FP mode profiles at 14.49 GHz, 14.49 GHz, 14.49 GHz and 14.435 GHz for selective y-variation of PHC slab with dimensions $8 \times 29$, $8 \times 50$, $8 \times 50$ and $8 \times 39$ layers, respectively. Arrows in (**d**–**g**) indicate source position.

The FP modes so far discussed in this work are single mode standing wave pattern. However by varying the PhC slab’s dimensions (both *x* and *y*), one can witness FP modes with interesting spatial profiles as shown in Fig. 5 for a point source with TE polarization. For example, a PhC slab with $12 \times 19$ layers supports two different FP modes; (1) TE_11_-like mode at 14.45 GHz (Fig. 5a), where the subscript 11 in TE_11_ refers the number of standing wave nodes in *x* and *y* directions respectively, and (2) TE_21_-like mode at 14.56 GHz. A PhC slab with $16 \times 15$ layers shows TE_31_-like mode at 14.61 GHz (Fig. 5c) (scanning profiles of these modes are given in SI Fig. S1 for further visualization).

In Fig. 5d–f, FP modes for specific *y*-variation of a PhC slab are shown, and the role of source position in exciting symmetric and anti-symmetric profiles is demonstrated. For instance, in Fig. 5d, a PhC slab consisting of $8 \times 29$ layers supports TE_11_-like mode at 14.49 GHz for a point source placed at the centre of the *y*-axis of the PhC slab. It is obvious to note that by nearly doubling the PhC layer along *y*-axis ($8 \times 50$ layers), an anti-symmetric TE_12_-like mode at 14.49 GHz with a maxima and minima can be expected as shown in Fig. 5e. However, the observed TE_12_-like mode corresponds to the source position indicated by the arrow in Fig. 5e–instead of an e-m source at the centre of the *y*-axis of a PhC slab. If a point source is placed at the centre of the *y*-axis, TE_12_-like mode formation is not complete as shown in Fig. 5f. This feature indicates that the source position influences the formation of FP modes in PhCs. In Fig. 5g, TE_13_-like mode at 14.435 GHz is witnessed for a PhC slab with $8 \times 39$ layers.

For *x*- and *y*-variation of PhC layers, it is found that all the FP mode frequencies correspond to the HLD regimes of dielectric PhCs and the mode formation depends on the point-source position.

### Comparison: FP mode in an ideal indefinite medium and in dielectric PhCs

**Figure 6:**
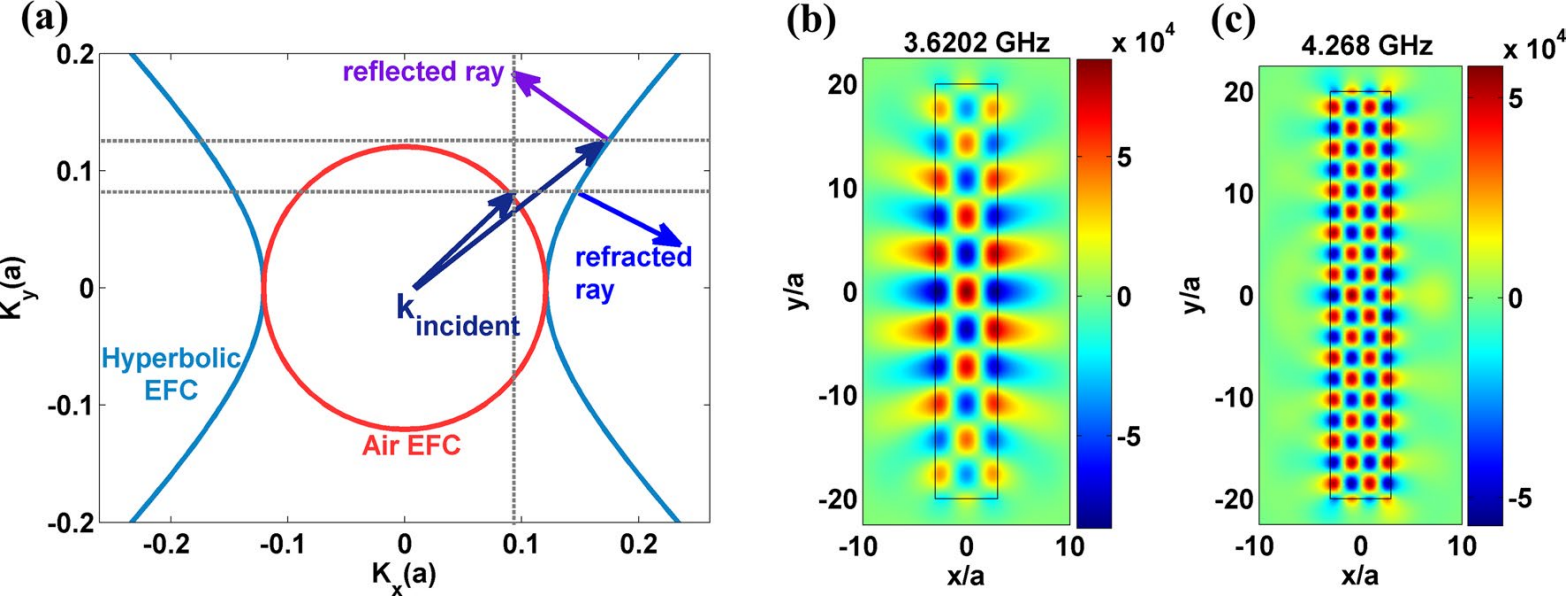
(**a**) Ray tracing in an *x*-hyperbolic EFC of an ideal indefinite slab at 3.6202 GHz. Circular EFC is corresponding to air medium at 3.6202 GHz. Refracted ray shows negative refraction and reflected ray shows internal reflection. (**b**,**c**) E_z_ field maps at 3.6202 GHz and 4.268 GHz for *x*-hyperbolic indefinite slab respectively.

In this section, FP mode formation in an ideal indefinite medium is presented for comparison with PhCs. An indefinite medium is modeled by considering an anisotropic slab for which the principal components $$\varepsilon = \left[ {\begin{array}{*{20}c} {\varepsilon_{xx} } & 0 & 0 \\ 0 & {\varepsilon_{yy} } & 0 \\ 0 & 0 & {\varepsilon_{zz} } \\ \end{array} } \right]{ , }\mu = \left[ {\begin{array}{*{20}c} {\mu_{xx} } & 0 & 0 \\ 0 & {\mu_{yy} } & 0 \\ 0 & 0 & {\mu_{zz} } \\ \end{array} } \right] \, $$ are not of same sign in all directions^14,15^. For simplicity, a magnetic type indefinite slab of dimension $7a \times 40a$ (‘*a*’ is the fundamental length-scale) is taken with the following parameters; $\mu_{x} = - 1, \, \mu_{y} = 1,{\text{ and }}\mu_{z} = 1$ and an isotropic relative permittivity value of 1 is assigned to the slab. These parameters will trace out an *x*-hyperbolic EFC in Fig. 6a. Ray tracing reveals that *x*-type hyperbolic EFC shows negative refraction for a TE wave incident from air medium. At the same time, for refraction from denser to rarer medium, a refracted ray at larger angle cannot be coupled to air medium (in Fig. 6a, construction line is not matched with air EFC for larger wavevectors). Hence side walls of an indefinite slab can act as a mirror for refracted rays inside the slab at larger angles. Secondly, by default, top/bottom walls of the *x*-hyperbolic indefinite slab acts as a mirror due to the non-availability of EFC. This feature offers internal reflection of e-m rays inside an indefinite slab and one can expect FP mode formation in it. In Fig. 6b,c shows two FP modes in *x*-hyperbolic indefinite slab at two different frequencies. Several work explored to this feature to build 3-D optical FP cavities^11^ and planar type magnetic FP cavity^13^.

The FP mode observed in dielectric PHCs at HLD frequencies is similar to the FP modes in an ideal indefinite slab. From these observations, two implications can be made; (1) partial focusing effect due to negative refraction in an indefinite slab was well-known^15^. When partial focusing is enabled with FP mode, near fields can be effectively transferred up to the image plane, as FP mode is localized at the slab–air interface. Similarly, in case of PhCs, the formation of FP modes at HLD regimes could also be harnessed for near field transfer. For instance in Wang and Kempa work^35^, interface of a PHC slab was modified by introducing disorders and similar FP mode was observed in focusing effect. Extending this study further with respect to the surface harnessing of PhC could be useful. (2) It is interesting to investigate how the FP mode will survive in a curved or a bending PhC configuration. This could be useful in realizing sensors for detecting bending angle and this task is attempted in next section.

### Bending angle sensor based on FP resonance in dielectric PhCs

**Figure 7:**
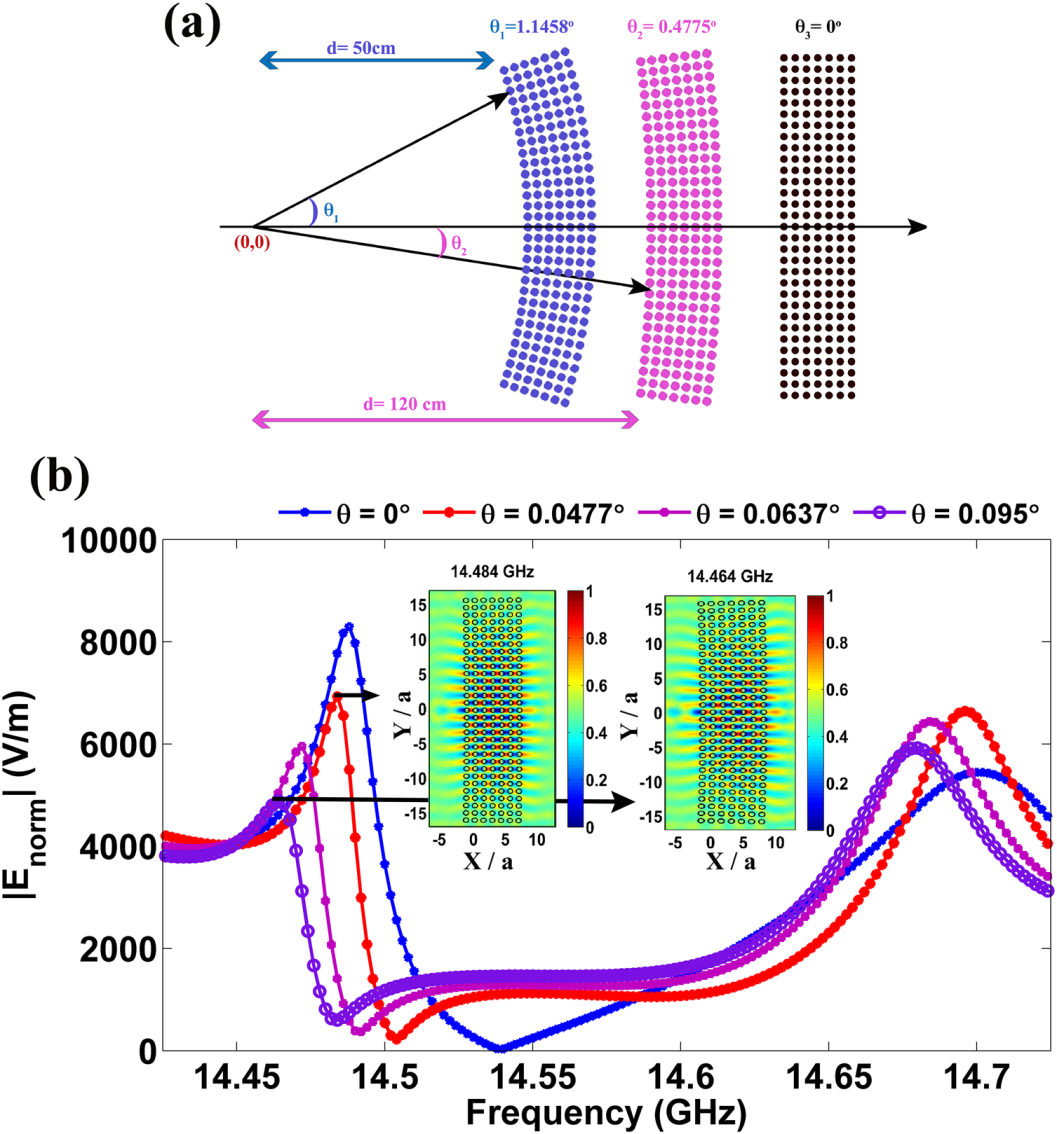
(**a**) PhC configurations with different bending angles; (**b**) FP resonance spectra correspond to various bending angles. In this figure, magnitude of the electric field detected on a point closer to the PhC wall is plotted. In the inset E_z_ field map is shown at 14.484 GHz and 14.464 GHz, which corresponds to bending angles of 0.0477° and 0.095°, respectively.

It is known that the interference of e-m waves due to reflection and refraction from a material is highly sensitive to the thickness variation, defect and roughness, as structural modification significantly alters the phase of the interfering beams. In the present study, we have observed that FP modes associated with HLD regimes in dielectric PhC are formed due to the interference of refracted beams at air–PhC interfaces. It is interesting to examine the role of bending in PhC on the formation of FP modes. In Fig. 7a, two different bending configurations ($\theta_{bend} = 1.1458^{ \circ }$ and $0.4775^{ \circ }$) are shown with respect to PhC without bending. Based on the characteristics of FP modes in PhC with bending, an angle sensor concept is demonstrated numerically.

In Fig. 7b, norm of the electric field monitored on a specific point near the edge of a PhC wall is plotted for various bending angles. It is noted that in the case of PhC without bending, two different resonances centered around 14.488 GHz and 14.7 GHz are observed. For angle sensing, anyone of these two frequencies can be monitored. When a bending angle is increased from $0^{ \circ }$ to $0.095^{ \circ }$, FP mode is shifted to lower frequency from 14.488 to 14.464 GHz. A bending angle of 0.0637° and 0.095° corresponds to the resonance shift of 0.016 GHz and 0.024 GHz, respectively. From this response, the angle sensitivity is deduced $\frac{{\Delta f_{{{\text{resonance}}}} }}{{\Delta \theta_{{{\text{bend}}}} }} = \frac{{f_{{r\left( {\text{with bending}} \right)}} - f_{{r\left( {\text{without bending}} \right)}} }}{{\theta_{{{\text{bending}}}} }}\sim 0.255{\text{ GHz per degree}}$. Apart from this linear regime, at very low bending angles, the shift in resonance is low, however, it is detectable. For instance, at 0.048°, a shift of 0.016 GHz is observed. If one keeps this as a limit, one can detect a bending angle at least less than 0.05° using FP modes. At an angle higher than 0.1°, the modes are degrading. It is also observed that apart from the shift in resonant frequency, the significant reduction in the intensity of FP mode (Fig. 7b) also can be used for sensing the bending angle.

### Bending angle sensing with finite-height 3-D PhC

**Figure 8:**
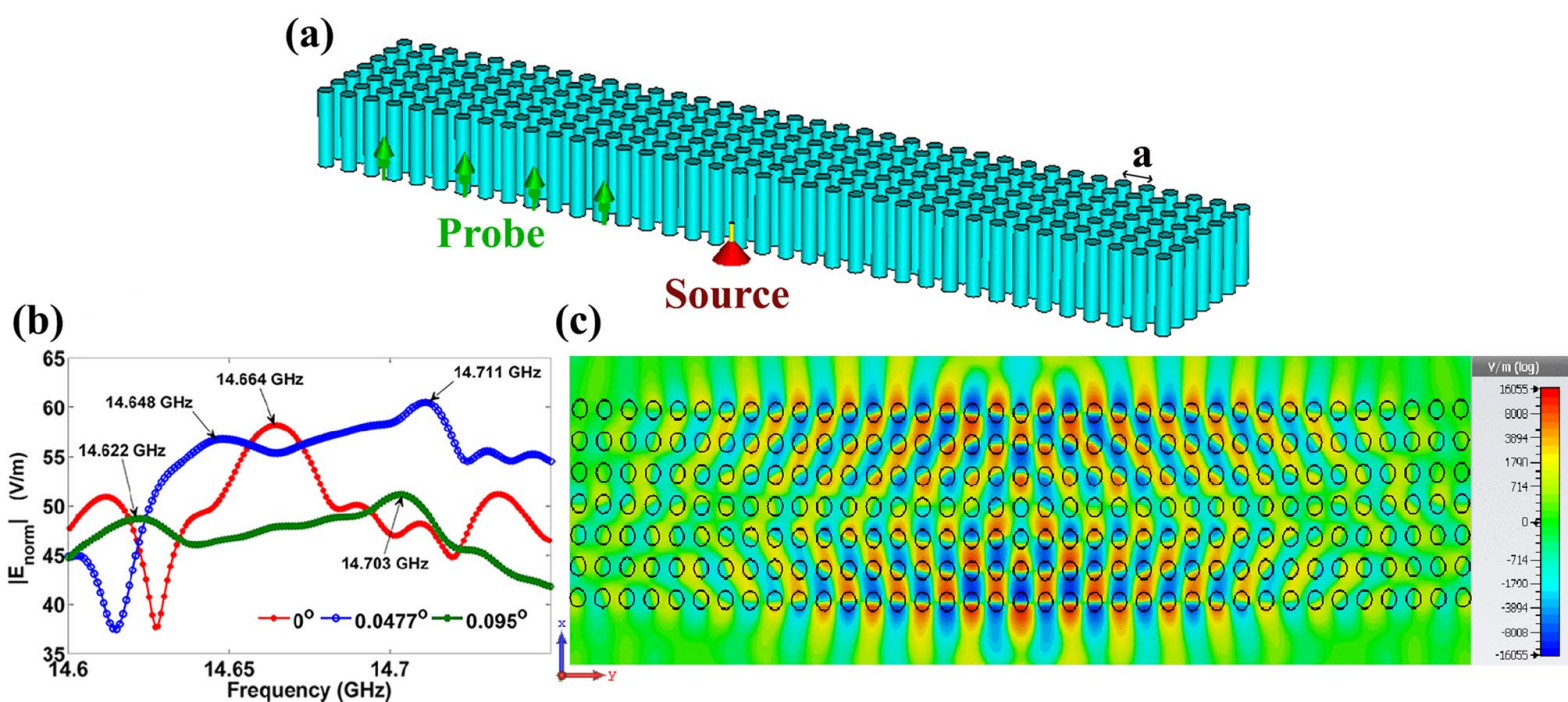
(**a**) 3-D configuration of a PhC with bending. Monopole source and probes are shown. (**b**) FP spectra for various bending angles. The mode peaks 14.664 GHz, 14.648 GHz and 14.622 GHz correspond to bending angles of 0°, 0.0477° and 0.095°, respectively. The other dominant peaks noted in the spectra also correspond to FP modes. (**c**) FP profile at 14.711 GHz for glass PhC with bending angle of 0.0477°.

The above investigation is restricted to 2-D computations with ideal conditions, in which the height of a PhC pillar is taken to be infinite and a theoretical point source is used in the formation of FP modes. However, for a practical realization, one will work with finite-height PhC and a practical point source. In this section, the results of 3-D full-wave e-m simulations are presented for a practical configuration shown in Fig. 8. Unlike infinite-height PhC, the setup shown in Fig. 8a suffers out-of plane radiation loss. However, from Fig. 8b,c one can verify that FP modes are existed for both bending and without bending cases. In Fig. 8b prominent peaks associated with FP mode resonances correspond to various bending angles. Particularly Fig. 8c corresponds to the PhC with a bending angle of 0.0477°. Due to out-of-plane radiation losses, red-shifted FP mode peaks are distorted in comparison with 2-D results. However, they are significant in Fig. 8b [14.664 GHz (0°), 14.648 GHz (0.0477°) and 14.622 GHz (0.095°)]. Hence including the limitations, the demonstrated bending angle sensing can be implemented successfully at microwave frequencies. Moreover, the FP modes in dielectric PhCs are scalable to terahertz and visible frequencies (Scaling results can be referred in SI Fig. S2).

## Conclusions

Fabry–Pérot modes associated with the HLD regimes of two-dimensional dielectric PhCs are investigated. The observed FP modes are formed due to the interference of refracted cylindrical TE wavefronts inside the PhC at HLD frequencies and they are surface localized at PhC-air interface. The interference formation is numerically verified for two line sources excited at an incident angle at HLD regimes. The projected bandstructure and EFC plot for PhCs reveal the existence of FP modes under three different cases namely (1) air EFC larger than HLD EFC, (2) air and HLD EFCs are intersecting and (3) HLD EFC is larger than air EFC. The effective index approach suggests that HLD regimes show mixed refractive behaviour in which group index is positive and phase index is negative. Though the critical angle cannot be easily derived for HLD regimes in PhCs due to its strong spatial dispersion characteristics, all three HLD EFCs provide larger group index values suitable for internal reflection conditions. The PhC slab of different dimensions supports interesting FP modes similar to TE_11_, TE_12_, TE_13_ modes at HLD frequencies. It is found that the source’s position is important in exciting the specific FP mode. The HLD regime of dielectric PhCs is compared with an ideal indefinite slab. The similar FP mode profile indicates that HLD regimes of dielectric PhC could be used for near field controlling and sensing application. For the application aspect, the sustainability of FP mode formation is studied with respect to varying the bending angle of PhCs. It is found that the shift in FP resonance can detect a bending angle as small as 0.05°. Finally, the bending angle sensing is tested with respect to the out-of-plane radiation losses using 3-D finite height PhC. As the reported FP modes in PhCs are scalable to other parts of the e-m spectra, bending angle sensing could be further extended to terahertz and optical domains.

## Methods

FP resonance spectra shown in Fig. 1 are obtained through two-dimensional full-wave e-m simulations using the finite-element method (FEM) based Comsol RF module^40^. Around the computational domain, low-reflecting boundary conditions (e.g., highly absorbing boundary conditions) are employed to mimic the open space. A TE point source is excited by setting up a line current of 1 A at a source point. A point probe is placed near the boundary of the PhC interface to detect the FP mode spectrum.

To obtain the projected band structure, open-source solver MIT Photonic Bands is used^41^. This e-m solver employs planewave expansion method and solves Maxwell’s Wave equation as a linear Hermitian eigenvalue problem. To solve projected bandstructure, super cell consists of $1 \times 11$ layers of glass rods arranged in a square lattice is taken. The dispersion relation is obtained by projecting out the *k*_*x*_ values within the I Brillouine zone, in the range of 0–0.5 (2π/a). The EFC plots shown in this work are obtained through MIT Photonic Bands. To solve EFC, single unit cell of square lattice PhC is taken and the band structure is solved within the k-grid defined with the range $\left( {k_{x} ,k_{y} } \right) \to 0{\text{ to }}0.5\left( {2\pi /a} \right)$. It may be noted that polarization definition is different in different e-m solvers. Throughout the work, direction of propagation is used as a reference to define the polarization. Hence TE mode is defined such that electric field is perpendicular (*y*) to the direction of propagation (*x*).

EFC plot is suffice to extract the phase index value. However the sign for phase index is determined through group-velocity calculations. MPB solver computes the group velocity components via the Hellman–Feynmann theorem as described in^42^.

Line sources used in the verification of interference due to refracted beams in the FP mode formation in Fig. 5, are created based on internal boundary conditions in COMSOL RF Module computations. Line sources are useful to create finite-size wavefront on a given length of line or curve. Line can be rotated about any given point so that the wave excitation at any incident angle can be easily modeled. The electric field boundary condition specified for a line source on a line is given as $\hat{n} \times \vec{E} = \hat{n} \times \vec{E}_{0}$ , where $\vec{E}_{0} = 1\hat{z} \, \left( {\text{V/m}} \right)$ is the initial TE polarized incident field and $\hat{n}$ is the unit normal vector.

Bending-angle sensing results of finite-height 3-D PhC is performed using finite-integration based commercial e-m solver CST Microwave Studio^43^. A glass PhC with a finite height of 1.45λ is taken in Fig. 8a. A copper wire with the length of λ/2 is excited with 1 A current source, which acts as a monopole TE polarization e-m source. The monopole source is kept behind the PhC slab at a distance of 0.146λ. Four point probe detectors are placed to probe the FP mode formation, as shown in Fig. 8a. Point probe detects the electric field at the probe position. Computationally solving a 3-D structure is challenging as the meshing of the structure influences accuracy of results and computational timings. In this calculation, 20 mesh cells per wavelength are kept.

## Supplementary information

Supplementary figure

## References

[1] Landreman PE, Chalabi H, Park J, Brongersma ML (2016). Fabry–Perot description for Mie resonances of rectangular dielectric nanowire optical resonators. Opt. Express.

[2] Frolov AY (2017). Near-field mapping of optical Fabry–Perot modes in all-dielectric nanoantennas. Nano Lett..

[3] Joannopoulos JD, Johnson SG, Winn JN, Meade RD (2008). Photonic Crystal Molding the Flow of Light.

[4] Ramakrishna SA (2005). Physics of negative refractive index materials. Rep. Prog. Phys..

[5] Susumu N, Baba T (2003). Roadmap on Photonic Crystals.

[6] Cui TJ, Smith DR, Liu R (2010). Metamaterials Theory, Design, and Applications, XXIII.

[7] Lalanne P, Sauvan C, Hugonin JP (2008). Photon confinement in photonic crystal nanocavities. Laser Photon. Rev..

[8] Shirai H, Ishii K, Miyagawa H, Koshiba S, Nakanishi S, Tsurumachi N (2014). Efficient terahertz emission, detection and ultrafast switching using one-dimensional photonic crystal microcavity. J. Opt. Soc. Am. B..

[9] Ameling R, Giessen H (2012). Microcavity plasmonics: Strong coupling of photonic cavities and plasmons. Laser Photon. Rev..

[10] Sadeghzadeh RA, Zarrabi FB (2016). Metamaterial Fabry–Perot cavity implementation for gain and bandwidth enhancement of THz dipole antenna. Optik.

[11] Yang X, Yao J, Rho J, Yin X, Zhang X (2012). Experimental realization of three-dimensional indefinite cavities at the nanoscale with anomalous scaling laws. Nat. Photon..

[12] Guo Z, Jiang H, Li Y, Chen H, Agarwal GS (2018). Enhancement of electromagnetically induced transparency in metamaterials using long range coupling mediated by a hyperbolic material. Opt. Express.

[13] Wang Y (2020). Circuit-based magnetic hyperbolic cavities. Phys. Rev. Appl..

[14] Smith DR, Kolinko P, Schurig D (2004). Negative refraction in indefinite media. J. Opt. Soc. Am. B..

[15] Smith DR, Schurig D, Mock JJ, Kolinko P, Rye P (2004). Partial focusing of radiation by a slab of indefinite media. Appl. Phys. Lett..

[16] Guo Z, Jiang H, Chen H (2020). Hyperbolic metamaterials: From dispersion manipulation to applications. J. Appl. Phys..

[17] Poddubny A, Iorsh I, Belov P, Kivshar Y (2013). Hyperbolic metamaterials. Nat. Photon..

[18] Bright TJ, Liu XL, Zhang ZM (2014). Energy streamlines in near-field radiative heat transfer between hyperbolic metamaterials. Opt. Express.

[19] Bogdanov AA, Suris RA (2012). Effect of the anisotropy of a conducting layer on the dispersion law of electromagnetic waves in layered metal-dielectric structures. JETP Lett..

[20] Kruk SS, Powell AD, Minovich A, Neshev ND, Kivshar YS (2012). Spatial dispersion of multilayer fishnet metaterials. Opt. Express.

[21] Simovski CR, Belov PA, Atrashchenko AV, Kivshar YS (2012). Wire metamaterials: Physics and applications. Adv. Mater..

[22] Iorsh IV, Mukhin IS, Shadrivov IV, Belov PA, Kivshar YS (2013). Hyperbolic metamaterials based on multilayer graphene structures. Phys. Rev. B.

[23] Yermakov YO, Ovcharenko IA, Song M, Bogdanov AA, Iorsh VI, Kivshar SYu (2015). Hybrid waves localized at hyperbolic metasurfaces. Phys. Rev. B Condens. Matter. Phys..

[24] Spinozzi E, Ciattoni A (2011). Ultrathin optical switch based on a liquid crystal/silver nanoparticles mixture as a tunable indefinite medium. Opt. Mater. Express.

[25] Sedighy HS, Guclu C, Campione S, Amirhosseini KM, Capolino F (2013). Wideband planar transmission line hyperbolic metamaterial for subwavelength focusing and resolution. IEEE Trans. Microw. Theory Tech..

[26] Narimanov EE (2014). Photonic hypercrystals. Phys. Rev. X.

[27] Foteinopoulou S, Soukoulis CM (2003). Negative refraction and left-handed behavior in two-dimensional photonic crystals. Phys. Rev. B Condens. Matter. Phys..

[28] Zhang X (2004). Absolute negative refraction and imaging of unpolarized electromagnetic waves by two-dimensional photonic crystals. Phys. Rev. B Condens. Matter. Phys..

[29] Foteinopoulou S, Soukoulis CM (2005). Electromagnetic wave propagation in two-dimensional photonic crystals: A study of anomalous refractive effects. Phys. Rev. B Condens. Matter. Phys..

[30] Luo C, Johnson SG, Joannopoulos JD, Pendry JB (2002). All-angle negative refraction without negative effective index. Phys. Rev. B Condens. Matter. Phys..

[31] Foteinopoulou S (2012). Photonic crystals as metamaterials. Phys. Rev. B Condens. Matter.

[32] Dominec F, Kadlec C, Němec H, Kužel P, Kadlec F (2014). Transition between metamaterial and photonic-crystal behavior in arrays of dielectric rods. Opt. Express.

[33] Meade RD, Brommer KD, Rappe AM, Joannopoulos JD (1991). Electromagnetic Bloch waves at the surface of a photonic crystal. Phys. Rev. B.

[34] Moreno E, García-Vidal FJ, Martín-Moreno L (2004). Enhanced transmission and beaming of light via photonic crystal surface modes. Phys. Rev. B Condens. Matter Mater. Phys..

[35] Wang X, Kempa K (2005). Effects of disorder on subwavelength lensing in two-dimensional photonic crystal slabs. Phys. Rev. B Condens. Matter Mater. Phys..

[36] Foteinopoulou S, Kafesaki M, Economou EN, Soukoulis CM (2007). Backward surface waves at photonic crystals. Phys. Rev. B Condens. Matter Mater. Phys..

[37] Lawrence FJ, Botten LC, Dossou KB, McPhedran RC, De Sterke CM (2010). Photonic-crystal surface modes found from impedances. Phys. Rev. A At. Mol. Opt. Phys..

[38] Yogesh N, Subramanian V (2011). Near field focusing effect and hyperbolic dispersion in dielectric photonic crystals. Prog. Electromagn. Res. M.

[39] Tang Z (2007). Optical properties of a square-lattice photonic crystal within the partial bandgap. J. Opt. Soc. Am. A.

[40] Whiteman, J. R. *The Mathematics of Finite Elements and Applications* (Wiley, Chichester, 1998). https://www.comsol.com.

[41] Johnson, S.G. and Joannopoulos, J.D. Block-iterative frequency-domain methods for Maxwell’s equations in a plane wave basis. *Opt. Express*, **8,** 173–190. https://ab-initio.mit.edu/mpb (2001). 10.1364/oe.8.000173 19417802

[42] Sakoda K (2005). Opitcal Properties of Photonic Crystals.

[43] CST Microwave Studio. www.cst.com.

