# Supplementary material for: Fabry–Pérot modes associated with hyperbolic-like dispersion in dielectric photonic crystals and demonstration of a bending angle sensor at microwave frequencies
Source: Sci Rep. 2020 Jul 6;10:11117. doi: 10.1038/s41598-020-67965-9 (PMC7338461; doi:10.1038/s41598-020-67965-9)
Supplement: Supplementary file 1 — Supplementary figure [file 41598_2020_67965_MOESM1_ESM.docx]

**Supplementary Figures of** “**Fabry-Pérot modes associated with hyperbolic-like dispersion in dielectric photonic crystals and demonstration of a bending angle sensor at microwave frequencies”**

**
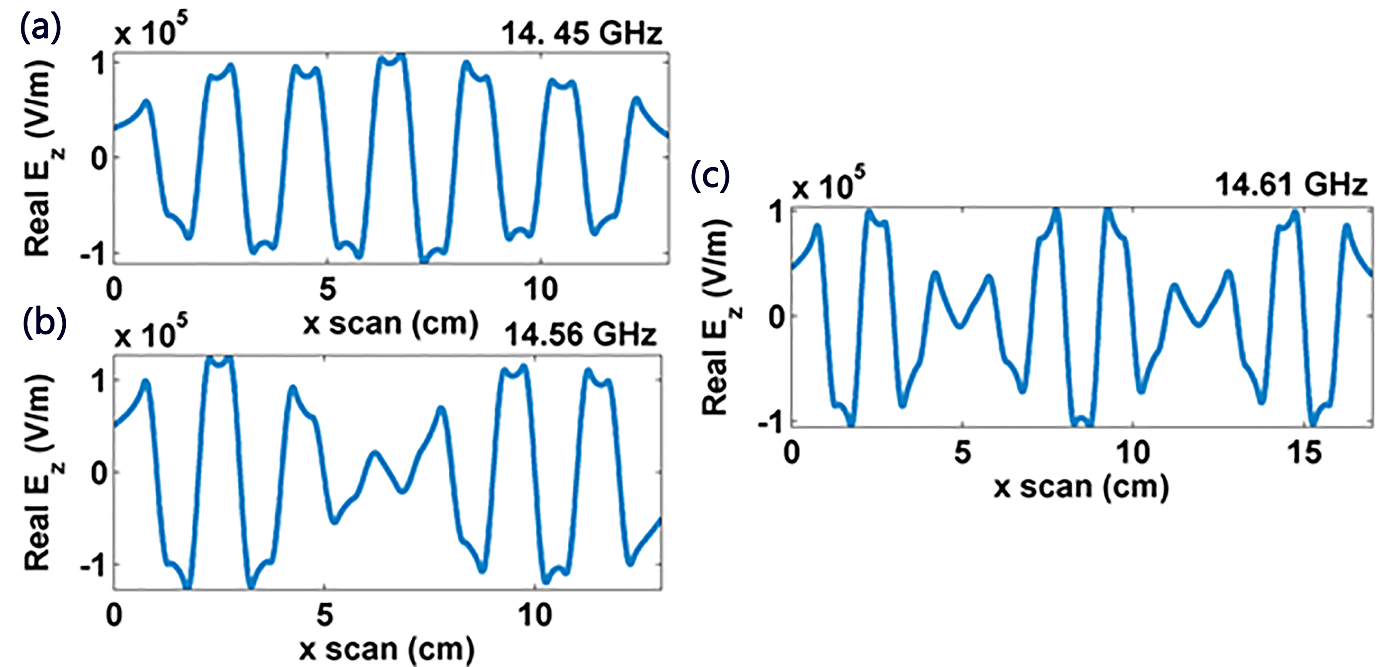
**

**Figure S1:** x-scanning of real part of E_z_ field profiles of Fig. 5(a) to Fig. 5(c) are shown in the plot. From the scanning one may observe (a) TE_11_-like mode at 14.45 GHz, (b) TE_12_- like mode at 14.56 GHz and (c) TE_13_-like mode at 14.61 GHz.


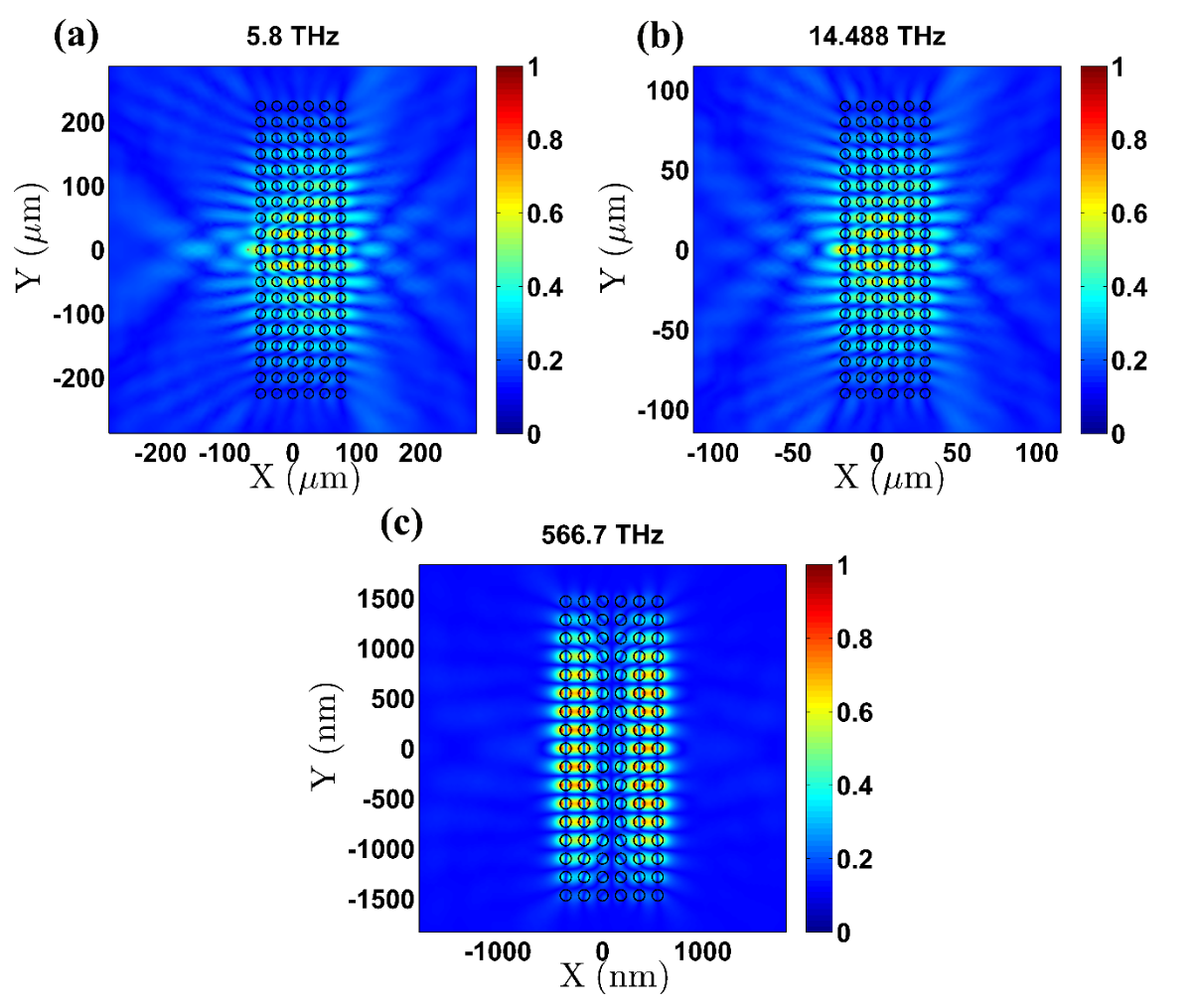


**Figure S2**. The norm of electric Field profile for glass (ε_r_ = 5.5) rods at THz frequency and a PhC made of a dielectric rod with ε_r_ = 12.96s for visible frequency shows the formation of FP mode with lattice constant (a) 25 µm (b) 10 µm and (c) 183.3 nm, respectively. The radius of the rod r = 0.3a was kept constant through the simulation in order to show the scalability.
